# Cancer prevalence and care disparities among individuals with intellectual disabilities: a cross-sectional pan-cancer analysis

**DOI:** 10.1016/j.esmorw.2025.100160

**Published:** 2025-06-25

**Authors:** T. Sappok, M.-L. Rosenbusch, R. Hering, M. Schulz, C. Kowalski, N.T. Sibert, T. Seufferlein, A.W. Berger

**Affiliations:** 1 Medical School and University Medical Center OWL, Bielefeld University, University Clinic for People with Neurodevelopmental Disorders, Mara Hospital, Bielefeld, Germany; 2 Central Research Institute of Ambulatory Health Care in Germany, Berlin, Germany; 3 Department of Health Services Research, German Cancer Society, Berlin, Germany; 4 Oncological Health Services Research, Department of Gynecology and Obstetrics, University Hospital Düsseldorf, Heinrich-Heine University Düsseldorf, Düsseldorf, Germany; 5 Ulm University Hospital, Department of Internal Medicine I, Ulm, Germany; 6 Evangelisches Krankenhaus Königin Elisabeth Herzberge, Clinic for Internal Medicine II - Gastroenterology and Gastrointestinal Oncology, Berlin, Germany

**Keywords:** intellectual disability, cancer, prevalence, health disparities, screening programmes

## Abstract

**Background:**

People with intellectual disability (ID) face considerable health disparities, with cancer being among the most frequent causes of premature death. A systematic analysis of the health care situation is necessary to further strengthen treatment and support for this highly vulnerable population.

**Patients and methods:**

In this cross-sectional study we analysed nationwide German outpatient health insurance data of 437 802 people with ID, which were compared to an age-, sex-, and district code-matched sample of people without ID.

**Results:**

Overall, people with ID (4.2% with cancer) showed lower odds ratios for a cancer diagnosis compared with the matched cohort without ID (5.1% with cancer) [C00-C97: odds ratio 0.83; 95% confidence interval 0.82-0.84; *P* < 0.0001]. Neoplasms of skin, gastrointestinal tract, and genital organs were most prevalent. People with ID less often attended cancer screening programs (OR 0.74; 0.74-0.75; *P* < 0.0001). Neoplasms of the brain, testicles, ovary, uterine body, and myeloid leukaemia occurred more often in people with ID (all *P* < 0.0001), while skin neoplasms, prostate cancer, tumours of the respiratory system, and breast cancer occurred less often (all *P* < 0.0001). People with ID and cancer were less often treated by specialists than matched controls.

**Conclusions:**

Difficulties in accessing the health care system and lower cancer screening rates may contribute to fewer cancer diagnoses. Our findings highlight specific cancer types—notably brain cancer, leukaemia, testicular and ovarian tumours—that show higher prevalence in individuals with ID compared with individuals without ID. These data underscore the increased vulnerability of the ID population to these particular malignancies, guiding future research, patient care, and screening efforts.

## Highlights

• The prevalence of cancer is lower in people with ID than in people without ID.
• Neoplasms of the brain, testicles, ovary, and leukaemia occur more frequently.
• Cancer screening is less common among people with ID than those without.
• People with ID and cancer often lack specialist care.
• People with ID and cancer were younger than those without ID and cancer.

## Introduction

Greater attention on the disparities in cancer care for individuals with intellectual disability (ID) is of paramount importance.^1^ According to the International Classification of Diseases (ICD)-11 (World Health Organization, 2022),^2^ ID are a group of etiologically diverse conditions that arise during the developmental period and are characterised by significantly below-average intellectual and adaptive functioning. Adaptive functioning pertains to how effectively an individual meets the standards of personal independence and social responsibility expected for their age and cultural group. It is evaluated across conceptual, social, and practical domains. People with intellectual disabilities generally require support in education, daily living activities, and social participation. This assistance is crucial for enhancing their quality of life and fostering their integration into society. People with an ID are faced with various health disparities and are at risk for premature death. Life expectancy for individuals with ID is reduced by ∼20 years compared to those without ID,^3^ with cancer (∼20%) being among the most frequent causes of death.^4^^,^^5^ Certain genetic syndromes predispose to cancer in this population.^6^ Obesity and smoking are two of the leading preventable risk factors for cancer, with recent studies indicating that obesity now causes more cases of certain cancers than smoking in some populations, whereas smoking remains the predominant risk factor for cancer mortality overall.^7^ These lifestyle factors may differ in people with ID and without ID and thereby influence the probability of certain cancer types.

However, the risk for cancer compared with the general population is still a matter of debate. A study based on a hospital care dataset from the Netherlands reported below-average incidence risks of people with ID (incidence rate ratio for male/female: 0.59/0.69).^8^ Cancer risk seems to be independent of the severity of ID, but may be more prevalent in syndromic causes of ID.^6^^,^^7^ Various studies described that cancer is particularly present in younger age groups and less prevalent in the elderly with ID compared with the general population.^6^^,^^7^^,^^9^ Accordingly, in a Swedish cohort study, the authors found an odds ratio (OR) of 0.63 in nearly 8000 people with ID aged >55 years compared with the general population.^10^ So far, no significant sex differences have been described concerning the overall cancer prevalence,^6^ with a slight preponderance of females over males.^7^, ^8^, ^9^

Elevated mortality rates have been reported for cancer patients with ID compared with the general population. A population-based retrospective cohort study from Canada, comprising a large data set of breast, colorectal, and lung cancer patients and reported adjusted hazard ratios of all-cause death of 2.74, 2.42, and 1.49 for breast, colorectal, and lung cancer patients with ID to those without ID.^10^ The findings were consistent for cancer-specific deaths and persisted with few exceptions regardless of stage at diagnosis.^11^ Recent data from the Netherlands and Scotland report increased standardised mortality ratios in people with ID and cancer compared with the general population.^4^^,^^12^ In a study on the mortality rates in England, the authors described a hazard ratio of 1.44 for neoplasm in a total of 16 666 patients with ID.^3^ The hazard ratio was similar for lung cancer in Korea (1.54-1.58), depending on the severity of ID.^11^

Thus, robust data on the relevant cancer types and the health care situation are warranted to further strengthen treatment and support in this highly vulnerable population. The following study therefore aims to give an overview of prevalences of various cancer types in people with ID and compared with the general population, the medical specialists currently taking care of people with ID, and the utilisation of cancer screening programs in this vulnerable patient group.

## Patients and methods

### Setting and design

Germany’s health care system features both statutory health insurance [insurance with income-dependent compulsory contribution up to a maximum income limit (income threshold), voluntary, and family insurance] and private (possible for high earners, self-employed, civil servants) insurance. The system offers comprehensive coverage with options between statutory and private insurance. Key features are mandatory insurance for all residents and free choice of doctors.

Outpatient physicians submit billing data to their regional Associations of Statutory Health Insurance (SHI; Kassenärztliche Vereinigungen) on a quarterly basis. The collected data include demographic information, International Classification of Diseases (ICD)-10 coded diagnoses, treatments, and procedures carried out. This system allows for comprehensive analysis of outpatient care for statutorily insured patients in Germany.

In a cross-sectional study design, nationwide outpatient data of the SHI according to § 295 SGB V (book V of the Social Code) covering ∼87.8% of the German population in 2019 were used to analyse the association between ID and various cancer types.^13^ In addition, risk factors for cancer [obesity (ICD-10: E66) and smoking (ICD-10: F17), the participation in cancer screening programs (fee schedule items of the doctor’s fee scale; EBM, Einheitlicher Bewertungsmaßstab) c.f. below], and the specialty of the practitioners were compared in people with and without ID.

The following cancer screenings were compared between people with and without ID:

• cervical cancer screening according to cancer screening guidelines
• prostate cancer screening for males
• test for occult blood (immunological faecal occult blood test)
• colonoscopy
• skin cancer screening
• mammography

All treating physicians and psychotherapists were included in the analysis. The following specialists are particularly relevant: general practitioners, paediatricians, anaesthesiologists, ophthalmologists, surgeons and orthopaedists, gynaecologists, ear, nose, and throat doctors, dermatologists, specialist internists (especially oncology, pulmonology, gastroenterology), neurologists, psychiatrists (including child and adolescent psychiatrists), radiologists, urologists, nuclear medicine specialists, radiation therapists, neurosurgeons, laboratory physicians, and pathologists.

### Selection process

Patients aged 0-107 years with at least one claim of benefits in 2019 (*N* = 72 229 371) were included in the study. People with ID were identified by a documented assured ICD-10 diagnoses (F7; F84.2; Q01-02; Q90-92; Q99.2) in at least two quarters in 2019 (M2Q). Controls neither had these inclusion diagnoses nor a diagnosis that may be associated with ID (F07; F84; G80, Q03-04; Q93-96; Q99). The entire study population comprised 438 028 patients with ID and 65 762 146 without ID. A control group for all patients with ID was determined using direct matching. For this purpose, the combinations of age in years, sex, and district code within the patients with ID were assigned sequential numbers that uniquely identify each combination (group).

**Figure 1:**
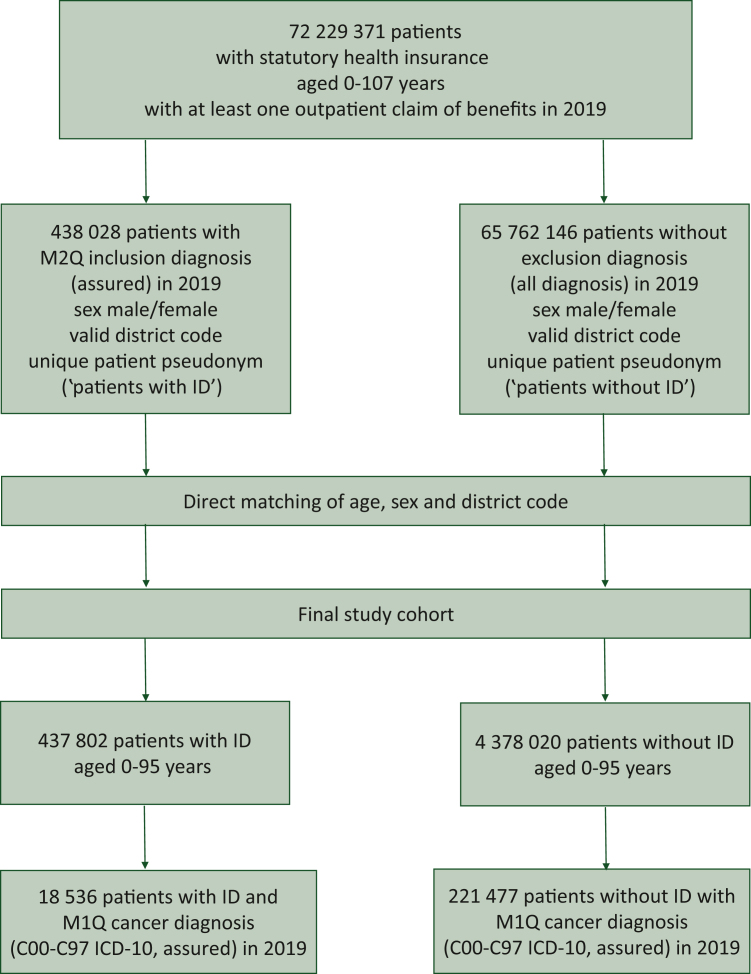
**Selection of patients with and without ID in 2019.** ID, intellectual disability; M2Q, minimum two quarters.

This group number was then assigned to the record containing the combination of age in years, sex, and district code for each patient with ID. Within each group, the rows were sorted randomly, and a sequential number was assigned to the patients within the group. The same table was created for the control group pool. Each patient with ID was matched to 10 patients without ID from the same group, using the numbering created within the groups. After matching, the final study population consisted of 437 802 people with and 4 378 020 without ID (age: 0-95 years). Details of the selection process are displayed in Figure 1.

### Statistical analysis

Applying a cross-sectional study design, we calculated univariate OR and 95% confidence intervals (CIs) using logistic regression (glm) (R package stats, R version 4.1.2) to estimate the association between ID and cancer occurrence for various cancer diagnoses. People with cancer were identified by a documented assured ICD-10 cancer diagnosis (C00-C97) in at least one quarter in 2019 (M1Q). For malignant neoplasms of the female and male genital organs (C51-C58; C60-C63) only female and male patients respectively were included in the calculations. Age distribution parameters were computed for different tumour entities in patients with and without ID. Estimates of the age difference in people with cancer—with and without ID—were calculated for various cancer diagnoses with the Mann–Whitney *U* test with 95% CI (Wilcox test) (R package stats, R version 4.1.2).

Univariate OR and 95% CI were calculated between patients with and without ID for obesity and smoking as risk factors for certain tumour types. Differences in participation in cancer screening programs was assessed for individuals with ID compared with the study participants without ID by calculating the univariate OR and their corresponding 95% CI. Among those with a cancer diagnosis, univariate OR and 95% CI were calculated to estimate the association between ID and treatment by physician specialty.

### Ethics

The study was approved by the ethics committee of the Berlin Medical Association (Eth-11/23).

## Results

### Study population

The final study population consisted of 437 802 patients with ID, including 18 536 patients with cancer (4.2%) and 4 378 020 patients without ID, including 221 477 with cancer (5.1%). There were 45.9% females and 54.1% males. Mean age was 39.4 years [standard deviation (SD) = 21.7], median age was 39 years. Due to the study design, no differences can be observed for age, sex, and district code between the two samples. Details of the study sample are given in Table 1.

**Table 1:** Characteristics of patients in the study sample

Characteristic	ID	Non-ID
**Total sample**	437 802	4 378 020
**Age (years)**		
Mean (SD)	39.4 (21.7)	39.4 (21.7)
Median	39	39
Min-max	0-95	0-95
**Sex**		
Male *n* (%)	236 740 (54.1)	2 367 400 (54.1)
Female *n* (%)	201 062 (45.9)	2 010 620 (45.9)
**Severity of ID**		
F70 (mild ID) *n* (%)	98 659 (22.5)	0
F71 (moderate ID) *n* (%)	73 590 (16.8)	0
F72 (severe ID) *n* (%)	42 465 (9.7)	0
F73 (profound ID) *n* (%)	16 005 (3.7)	0
**F74 (dissociated ID), F78 (other ID), F79 (unspecified ID) *n* (%)**	147 544 (33.7)	0
**Other inclusion diagnoses (severity of ID not determinable: congenital malformations, deformations and chromosomal abnormalities: Q01; Q02; Q90; Q91; Q92; Q99.2)** and **F84.2 (Rett syndrome) *n* (%)**	59 539 (13.6)	0
Total *n* (%)	437 802 (100)	0
**Risk factors**		
**Obesity (E66; % with cancer diagnosis)**	85 751 (19.59)	484 154 (11.06)
**Smoking (F17; % with cancer diagnosis)**	26 422 (6.04)	275 368 (6.29)

### Prevalence for several cancer types in people with and without ID

**Figure 2:**
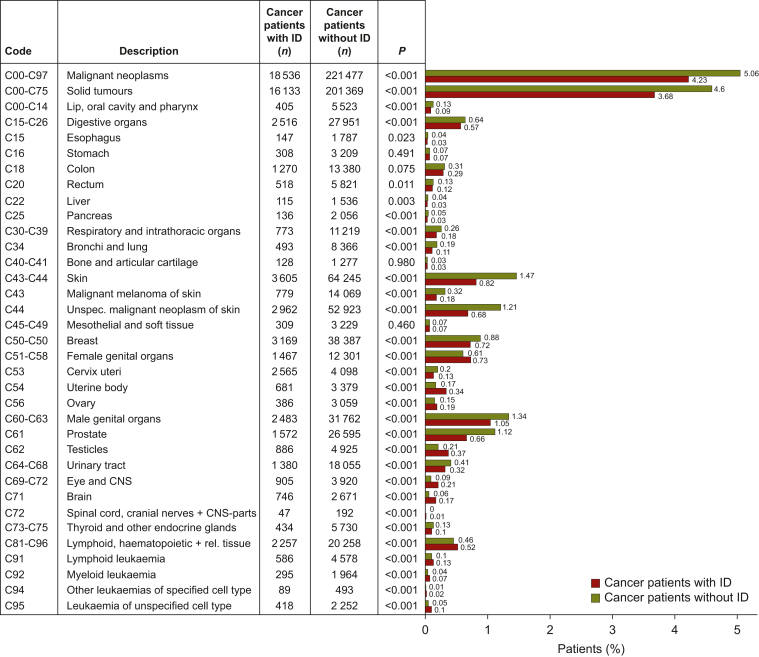
**Relative frequencies (%) of several cancer types in people with intellectual disability (ID) (red bars) and people without ID (green bars).** Tumour types with significant univariate odds ratio (OR) (using logistic regression) are printed in bold letters. CNS, central nervous system; rel., related, unspec., unspecified.

The relative frequencies (in %) of various cancer types are displayed in Figure 2 for people with ID (red bars) and people without ID (green bars).

Regarding the relative frequencies in people with ID, the most prevalent cancer types were breast cancer (C50), malignant neoplasms of the skin (C43-44), malignant neoplasms of lymphatic, haematopoietic and related tissue (C81-96), malignant brain tumours (C71) and malignant neoplasms of the urinary organs (C64-C68). In addition, for females, malignant neoplasms of the uterine body (C54) and ovary (C56), and for males, prostate cancer (C61) and cancer of the testis (C62) were highly prevalent in the respective group.

### Differences in cancer types in people with and without ID

**Figure 3:**
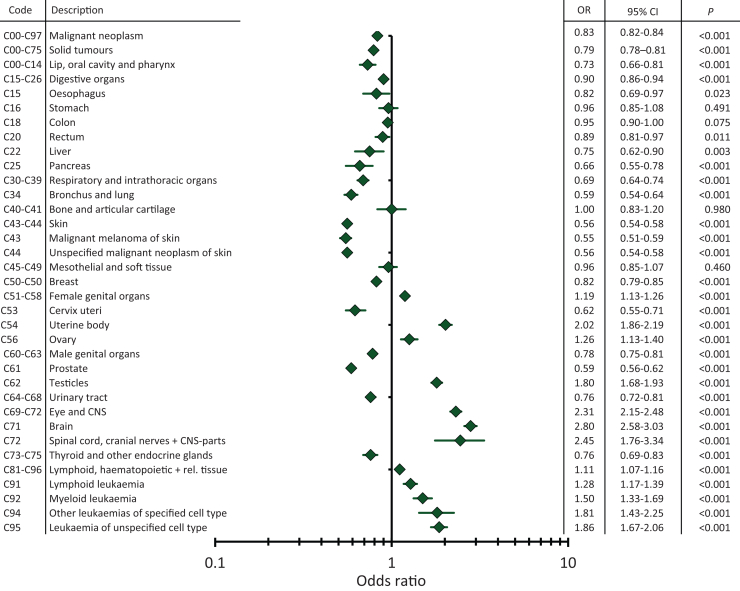
**Forest plot displaying the odds ratio (OR) and 95% confidence interval (CI) (bars) for several cancer entities in people with intellectual disability (ID) compared with people without ID.** ORs for cancer types with higher odds documented in people with ID can be seen on the right, whereas those more prevalent in people without ID are displayed on the left. Tumour types with significant OR in people with and without ID are printed in bold letters. CNS, central nervous system; rel., related.

People with ID showed lower odds for a documented cancer than those without ID (OR 0.83, 95% CI 0.82-0.84, *P* < 0.0001 for all data) (Figure 3). Certain cancer types occurred more often among people with ID, such as malignant neoplasms of the brain (OR 2.80, 95% CI 2.58-3.03, *P* < 0.0001), other parts of the central nervous system (OR 2.45, 95% CI 1.76-3.34, *P* < 0.0001), the testicles (OR 1.80, 95% CI 1.68-1.93, *P* < 0.0001), the ovary (OR 1.26, 95% CI 1.13-1.4, *P* < 0.0001), the uterine body (OR 2.02, 95% CI 1.86-2.19, *P* < 0.0001), leukaemia of unspecified cell type (OR 1.86, 95% CI 1.67-2.06, *P* < 0.0001) and other leukaemia of specified cell type (OR 1.81, 95% CI 1.43-2.25, *P* < 0.0001). However, other entities such as malignant melanomas (OR 0.55, 95% CI 0.51-0.59), prostate cancer (OR 0.59, 95% CI 0.56-0.62, *P* < 0.0001), tumours in the respiratory system (OR 0.69, 95% CI 0.64-0.74, *P* < 0.0001) and breast cancer (OR 0.82, 95% CI 0.79-0.85, *P* < 0.0001) occurred less often among people with ID.

### Age differences in people with and without ID

**Figure 4:**
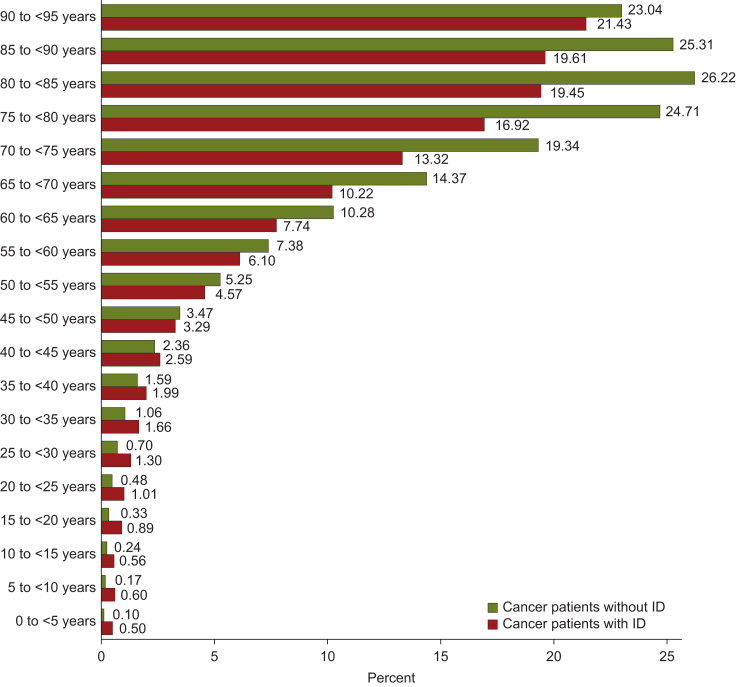
**Distribution of age in people with cancer with and without intellectual disability (ID) across ages (years).** The bars represent the proportion (%) of patients with cancer (C00-97) in the respective age group.

Despite the matching for age in the total sample, people with ID and cancer were younger than those without ID across all cancer types [C00-C97: mean 60.0 (SD = 17.9) versus 63.8 (SD = 14.6) years, median 62 versus 65 years, *P* < 0.0001] and in the solid tumours [C00-C75: 61.5 (SD = 16.4) versus 64.4 (SD = 14.1) years, median 63 versus 65 years, *P* < 0.0001]. In younger ages, the documented cancer diagnoses predominated in people with ID, whereas after the age of 45 years, cancer was more frequent in those without ID (Figure 4).

### Age differences according to cancer type

**Figure 5:**
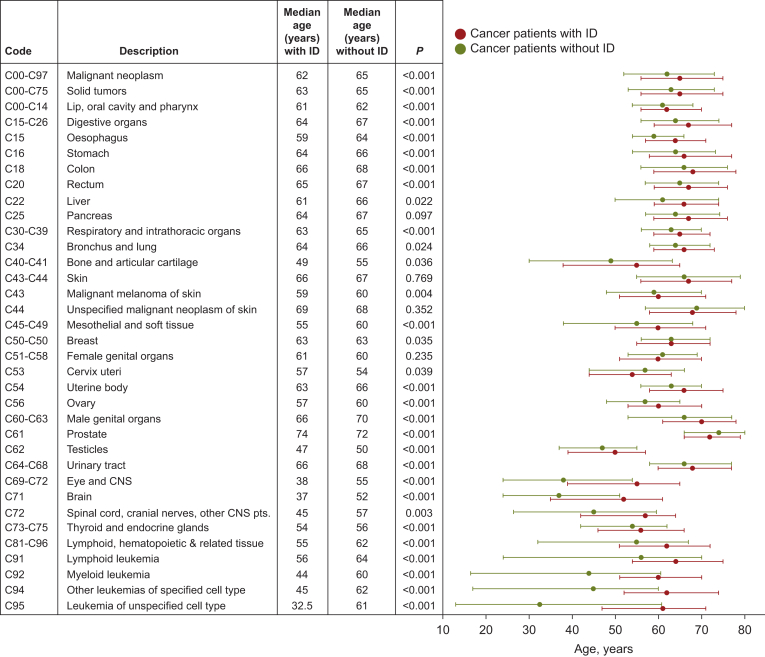
**Mean and median ages (years) for different tumour entities in people with and without intellectual disability (ID).** The graph displays the median ages including the quartiles (1. and 3.) for people with (in red) and without ID (in green). Tumour types with significant differences in age are printed in bold letters. CNS, central nervous system; pts., parts.

The Mann–Whitney *U* test showed no significant age differences for malignant melanoma (C43-C44: 65.2 versus 65.8 years, *P* = 0.7692) and breast cancer (C50: 64.1 versus 63.6 years, estimator: 0, *P* = 0.0352), whereas average age differences of ∼10 years were observed for brain (C71: 37.8 versus 48.6 years, *P* < 0.0001) and blood (C81-96: 49.7 versus 59.4 years, *P* < 0.0001) neoplasms. Figure 5 displays the median ages including the quartiles (1. and 3.) for different tumour entities in people with and without ID.

### Care specialties

People with ID and a cancer diagnosis (C00-C97) more often consulted a general practitioner or neurologist/psychiatrist, whereas those without ID were more often treated by the respective specialist such as surgeons, specialists for internal medicine, oncology, gastroenterology, dermatology, etc. Details are given in Supplementary Figure S1, available at https://doi.org/10.1016/j.annonc.2024.06.010.

### Risk factors and screening for cancer in people with and without ID

ID was associated with increased odds for obesity (E66: OR 1.96, 95% CI 1.94-1.97, *P* < 0.0001), while the odds for tobacco smoking were only slightly below those for people without ID (F17: OR 0.96, 95% CI 0.94-0.97, *P* < 0.0001). People with ID were less likely to attend cancer screening programmes (OR: 0.74, 95% CI 0.74-0.75, *P* < 0.0001). ORs and 95% CIs for individual types of screening are shown in Table 2.

**Table 2:** Cancer screening in people with and without ID

Description	Code	Patients with ID *n* (%)	Patients without ID *n* (%)	Odds ratio	95% CI	*P*-value
Overall cancer screenings		107 347 (24.52)	1 329 350 (30.36)	0.74	0.74-0.75	<0.0001
Cervical cancer screening for females according to cancer screening guidelines	01730	59 517 (13.59)	812 504 (18.56)	0.69	0.68-0.70	<0.0001
Prostate cancer screening for males	01731	22 271 (5.09)	224 960 (5.14)	0.99	0.98-1.00	0.1418
Test for occult blood (iFOBT)	01737 and 01738	13 419 (3.07)	193 510 (4.42)	0.68	0.67-0.70	<0.0001
Colonoscopy	01741	961 (0.22)	31 356 (0.72)	0.30	0.29-0.32	<0.0001
Skin cancer screening	01745 and 01746	31 669 (7.23)	391 327 (8.94)	0.79	0.78-0.80	<0.0001
Mammography	01750	10 793 (2.47)	158 509 (3.62)	0.67	0.66-0.69	<0.0001

CI, confidence interval; ID, intellectual disability; iFOBT, immunological faecal occult blood test.

## Discussion

People with ID utilizing the outpatient health care system showed lower prevalence for cancer, earlier age of onset, more often consulted a general practitioner or neurologist/psychiatrist and less often attended cancer screening programmes compared with people without ID.

Our study is in line with several other reports showing younger ages of cancer onset in people with ID compared with the general population. Despite matching for age in the overall sample, cancer patients with ID were on average 4 years younger than cancer patients without ID (60 versus 64 years). Age differences in favour of younger ages for people with ID were particularly distinctive in neoplasms of the brain and the blood. To the best of our knowledge, this is the first study describing these age differences for tumours of the central nervous system in people with ID. In line with our data, several studies showed increased risk for malignant brain tumours in people with ID compared with the general population.^6^, ^7^, ^8^ Further research is necessary to explore the pathogenetic mechanisms leading to the increased prevalence and younger ages of onset in the ID population. Also for leukaemia, younger ages and increased odds could be observed in the ID population, which may be driven by people with Down syndrome.^14^ Significant progress has been made in identifying genes on chromosome 21 and possible mechanisms for driving leukaemogenesis, which increased our understanding of the disease and resulted in adapted treatment regimens (for review, c.f. Baruchel et al., 2023).^15^ In the future, research may be expanded to people with leukaemia and ID not caused by Down syndrome to examine the underlying mechanisms and optimise therapy plans.

People with ID showed lower overall odds for a documented cancer diagnosis than those without ID. The study population comprises 18 536 persons with ID and cancer. This is the largest sample ever analysed for cancer in ID. The lower rates for cancer care are in line with a study by Cuypers et al. (2020) who found cancer incidence rates of 0.64 for people with ID, which may be due to under-diagnosis and undertreatment of cancer.^8^ A Swedish cohort study from 1974-2013 comprising ∼3.5 million people in total and ∼28 000 people with ID (188 cancer cases; incidence rate 62/1000 person-years) found increased hazard ratios for cancer incidence in ID (1.57),^16^ whereas a Finnish^17^ (*N* = 2173 people with ID; 173 cancer cases in 30 years) and an Australian^18^ (*N* = 9409 people with ID; 200 cancer cases) cohort study described equal standardised incidence ratios for cancer compared with the general population of 0.9 and 1.01 (females)/1.14 (males), respectively. In a recent review comprising 55 articles on the prevalence of cancer in people with ID, the authors conclude that the overall cancer risk is lower or comparable with the general population, whereas specific genetic syndromes such as Down syndrome may increase the risk for certain cancer types.^19^

Another possible explanation for the reduced cancer prevalence in our sample may be that patients with ID have had lower survival rates and hence appear in these cross-sectional health insurance data analyses for a shorter period and therefore less frequently than the cancer survivors without ID, who may live longer. This hypothesis is supported by data from Heslop et al. (2022) who found that people with ID are frequently diagnosed in stage IV of the cancer disease.^18^ ID may be an under-recognised driver of cancer mortality.^20^ Individuals with ID may experience higher mortality rates due to being diagnosed with cancer at more advanced stages.^20^ Additionally, complicated access to the health care system and differences in cancer treatment decision-making may further contribute to these increased mortality rates in people with ID.^21^ For several cancer types, consistently lower rates for surgery, chemotherapy, or radiotherapy were described for people with ID who were diagnosed with cancer.^14^^,^^22^ Tosetti and Kuper (2023) summarised various reasons that may lead to the disparities in cancer care, such as poorer quality of cancer care, poorer access to state-of-the-art care or curative therapies, delays in treatment, undertreatment or overly invasive treatment, poorer access to inpatient services, reduced utilisation of specialists, and inadequate quality of care at the end of life.^23^ Additional problems such as barriers in communication, lack of training and knowledge of clinical staff, but also discriminative attitudes and wrong assumptions further increased poorer outcomes. In many cases, cancer is diagnosed as an incidental finding during an emergency. Persons with ID participate less frequently in screening programmes.^18^^,^^24^

Our study helps to focus future research, but also patient care and potentially screening of certain cancer types that occur more often in people with ID compared with the general population such as brain cancer, leukaemia, and germ cell tumours (testicle and ovary). These results are in line with other cross-sectional studies, indicating a higher vulnerability of people with ID for these diseases compared with the general population.^6^, ^7^, ^8^ There may be a common genetic aetiology for the respective cancer types and ID. Certain forms of ID, notably Rett Syndrome and Down Syndrome, are associated with a chronic inflammatory state, which may contribute to poor defence mechanisms against cell damage and thereby increase the risk for brain cancer.^25^, ^26^, ^27^ Other cancer types that occurred more often were cancers of the uterine body. This was already seen in smaller study samples and may be mediated by obesity.^6^^,^^7^

Interestingly, for certain entities such as malignant melanomas, prostate cancer, tumours in the respiratory system, breast, cervix uteri, the urinary tract, and the thyroid, the OR indicated relatively lower rates in people with ID. Lower prevalence for tumours of the lung, skin, breast, cervix uteri, and the prostate have been consistently reported in various other studies.^6^, ^7^, ^8^^,^^10^ For tumours of the respiratory and the urinary system, this could be related to lifestyle factors such as lower smoking rates, which could also be observed in our sample. The reduced risk for malignant skin cancers may be related to lower sun exposure in persons with ID. Lower sexual activity may account for lower rates of cervix carcinoma. The lower prevalence of cervical cancer observed among individuals with ID may, paradoxically, be attributed to reduced participation in cervical screening programs within this population.^28^ This underscores the complex interplay between health care access, preventive measures, and cancer detection rates in vulnerable groups. For certain tumour types, the prevalence studies found inconclusive results. Although we observed lower OR for the prevalence for tumours of the thyroid gland among persons with ID, in contrast Patja et al. (2001) found increased SIR for ID. Sullivan et al. (2004) report higher SIR for males (1.69) than for females (0.82) with ID compared with the general population. Thus, further factors including sex, age, and syndromic ID may need to be considered for thyroid cancers. This may also apply to tumours of the gastrointestinal tract. While we found slightly decreased odds for tumours of the gastrointestinal tract, others described lower or higher rates in ID versus the general population.^6^^,^^10^^,^^29^ In people with profound and multiple disabilities, cancers of the digestive tract occur earlier and more frequently, and age and severity of ID need to be considered when evaluating individual health risks.^23^ Accordingly, in an analysis of deceased adults with ID in England, Heslop et al. (2022) found that almost half of those who died from a gastrointestinal cancer were below the age threshold for colorectal screening.^20^

Despite the lower prevalence for cancer of the breast, skin, colon, and prostate in people with ID compared with those without ID, these entities were among the top 10 cancer types in people with ID. Screening for these cancers remains equally important in people with ID. However, as could be seen in our data, people with ID participate less frequently in cancer screening programmes than those without ID (OR 0.74). Pooling these screening estimates presents significant challenges due to considerable heterogeneity among cancer sites, as each type of cancer possesses distinct biological characteristics and screening implications. Additionally, differences in invasiveness and methodology among screening tests introduce further variability, making direct comparisons and synthesis of results more complex. For the United States and Korea, even lower adjusted OR were found for mammography screening in ID versus the general population (0.63 and 0.403, respectively).^28^^,^^30^Also in other countries, mammography screening was applied less often in people with ID versus the general population (25% versus 62% in Denmark; 35% versus 55% in Australia).^24^^,^^31^ These health disparities are also true for pap smears (adjusted [a]OR 0.17) and colorectal cancer screening by a faecal occult blood test (aOR 0.61).^24^^,^^32^ Noninvasive screening measures such as the faecal occult blood test may be applicable in people with adherence difficulties or increased risks associated with colonoscopy. Miyashita et al. (2024) suggested using ultrasonography for breast cancer screening for females >30 years old, especially for those with severe motor and intellectual disabilities.^33^

In our secondary data analysis, people with ID and a documented diagnosis of cancer more often consulted a general practitioner or neurologist/psychiatrist, whereas those without ID were more often treated by the respective specialist. Within the data set, we could only analyse associations, thus, people with ID and cancer may have consulted a neurologist or a psychiatrist due to another neurological or psychiatric disorder and not because of their cancer diagnosis. However, other studies also reported lower rates of specialist health care utilisation in people with ID.^21^ Moreover, in an analysis from England, more than one-third of cancer diagnoses (35%) occurred via emergency presentations and almost half of cancers were at stage IV when diagnosed.^18^

Several limitations need to be considered when interpreting the results. A significant limitation of our study lies in the utilisation of ICD-10 coding for identifying individuals with ID. This methodology, while standardised, may not capture the full spectrum of ID cases, potentially leading to an underestimation of the true prevalence. The nuanced nature of ID, coupled with potential variations in diagnostic practices, suggests that a proportion of cases may elude detection through this coding system alone. Due to the cross-sectional study design, we examined associations, not causal relationships, and only prevalences, not incidence rates. We had no information regarding diagnoses and treatment in the inpatient sector, no cancer stages, and no mortality rates. The lack of information for inpatient data may lead to an underreporting of severe cases who may directly be admitted to a hospital without consultation of an outpatient doctor.^18^ We have no information as to why a person visited a doctor; whether it was due to the cancer diagnosis or due to another health reason. The interpretation of age disparities in our study is constrained by the methodological decision to age-match the overall ID cohort with the non-ID population. This approach, while enhancing comparability across other variables, introduces a potential underestimation of age-related differences in cancer onset. The observed age discrepancies in specific cancer types, notably those affecting the central nervous system and haematological malignancies, may in fact be more pronounced in an unmatched, naturalistic sample. Our age-matched design, while methodologically robust, may inadvertently attenuate the full extent of these age-related disparities. Therefore, the data on the consultations need to be interpreted with caution. However, the strength of our analysis lies in the consideration of nationwide outpatient claims data of patients with statutory health insurance in Germany.

Given the heterogeneous nature of ID, encompassing a diverse array of aetiologies and severity levels, there is a compelling need for more nuanced investigations into cancer screening practices and prevalence within this population. Future research should therefore aim to stratify ID subgroups based on aetiology and severity to examine potential variations in cancer risk and screening uptake. Differential analyses of cancer screening rates across various ID subpopulations should be conducted, identifying potential disparities and barriers to access. The interplay between genetic factors associated with certain forms of ID and cancer susceptibility needs to be examined. Future research endeavours may benefit from employing both matched and unmatched analyses to provide a more comprehensive understanding of the age- and sex-related nuances in cancer onset between ID and non-ID populations. More nuanced data collection methodologies that capture the primary reasons for medical consultations could significantly enhance our understanding of health care utilisation patterns in this vulnerable population. Longitudinal studies are essential for elucidating temporal trends and patterns over extended periods, enabling the identification of developmental trajectories and causal relationships that may not be apparent in cross-sectional analyses. This proposed research agenda would significantly enhance our understanding of cancer dynamics within the ID population, potentially leading to more effective, personalised screening and prevention strategies.

This study offers robust, population-based evidence that individuals with ID in Germany are less likely to receive cancer diagnoses and participate in cancer screenings compared with the general population. By identifying specific cancer types-such as brain, leukaemia, testicular, and ovarian cancers that are either more prevalent or underdiagnosed among people with ID, our research highlights critical gaps in cancer care for this vulnerable group. These findings emphasise the urgent need for targeted interventions, improved access to health care, and tailored cancer screening programs to reduce health disparities and enhance outcomes for individuals with intellectual disabilities. In summary, our results demonstrate that, although medical services are available, additional educational support and outreach are essential to ensure that people with ID are effectively reached, and equitable, guideline-based cancer screening and treatment are achieved.

## Declaration of generative AI and AI-assisted technologies in the writing process

During the preparation of this work the author(s) used perplexity.ai (https://www.perplexity.ai) to refine the English language. After using this tool/service, the author(s) reviewed and edited the content as needed and take(s) full responsibility for the content of the publication.

## Funding

No funding received.
