# Supplementary material for: Cancer prevalence and care disparities among individuals with intellectual disabilities: a cross-sectional pan-cancer analysis
Source: ESMO Real World Data Digit Oncol. 2025 Jun 25;9:100160. doi: 10.1016/j.esmorw.2025.100160 (PMC12836748; doi:10.1016/j.esmorw.2025.100160)
Supplement: Supplementary Figure S1 [file mmc1.pptx]

## Slide 1
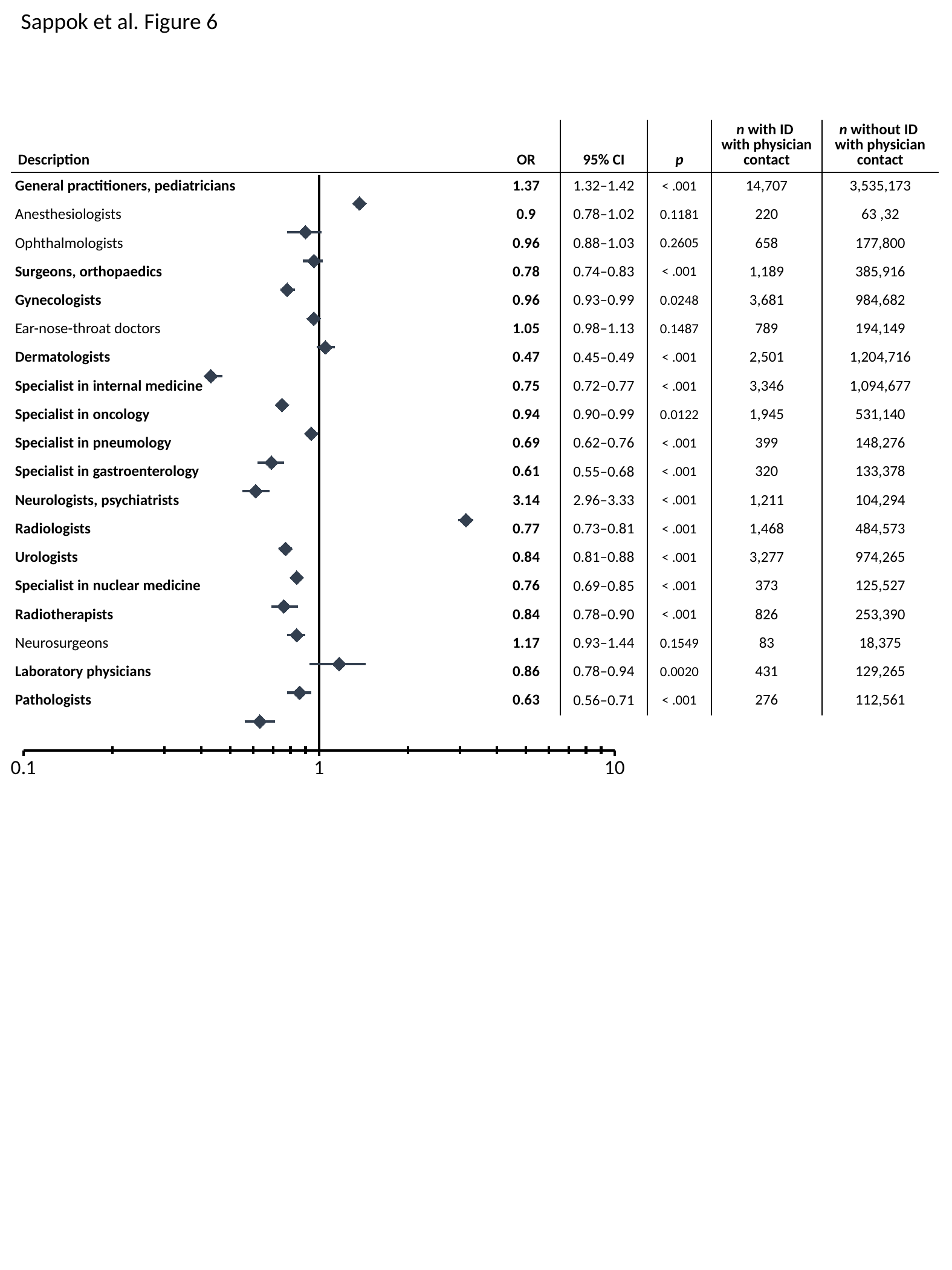

Sappok et al. Figure 6
| Description | | OR | 95% CI | p | n with ID with physician contact | n without ID with physician contact |
| --- | --- | --- | --- | --- | --- | --- |
| General practitioners, pediatricians | | 1.37 | 1.32–1.42 | < .001 | 14,707 | 3,535,173 |
| Anesthesiologists | | 0.9 | 0.78–1.02 | 0.1181 | 220 | 63 ,32 |
| Ophthalmologists | | 0.96 | 0.88–1.03 | 0.2605 | 658 | 177,800 |
| Surgeons, orthopaedics | | 0.78 | 0.74–0.83 | < .001 | 1,189 | 385,916 |
| Gynecologists | | 0.96 | 0.93–0.99 | 0.0248 | 3,681 | 984,682 |
| Ear-nose-throat doctors | | 1.05 | 0.98–1.13 | 0.1487 | 789 | 194,149 |
| Dermatologists | | 0.47 | 0.45–0.49 | < .001 | 2,501 | 1,204,716 |
| Specialist in internal medicine | | 0.75 | 0.72–0.77 | < .001 | 3,346 | 1,094,677 |
| Specialist in oncology | | 0.94 | 0.90–0.99 | 0.0122 | 1,945 | 531,140 |
| Specialist in pneumology | | 0.69 | 0.62–0.76 | < .001 | 399 | 148,276 |
| Specialist in gastroenterology | | 0.61 | 0.55–0.68 | < .001 | 320 | 133,378 |
| Neurologists, psychiatrists | | 3.14 | 2.96–3.33 | < .001 | 1,211 | 104,294 |
| Radiologists | | 0.77 | 0.73–0.81 | < .001 | 1,468 | 484,573 |
| Urologists | | 0.84 | 0.81–0.88 | < .001 | 3,277 | 974,265 |
| Specialist in nuclear medicine | | 0.76 | 0.69–0.85 | < .001 | 373 | 125,527 |
| Radiotherapists | | 0.84 | 0.78–0.90 | < .001 | 826 | 253,390 |
| Neurosurgeons | | 1.17 | 0.93–1.44 | 0.1549 | 83 | 18,375 |
| Laboratory physicians | | 0.86 | 0.78–0.94 | 0.0020 | 431 | 129,265 |
| Pathologists | | 0.63 | 0.56–0.71 | < .001 | 276 | 112,561 |
### Chart
| Category | | |
|---|---|---|
